# Nomograms for Predicting the Prognostic Value of Pre-Therapeutic CA15-3 and CEA Serum Levels in TNBC Patients

**DOI:** 10.1371/journal.pone.0161902

**Published:** 2016-08-25

**Authors:** Danian Dai, Bo Chen, Hailin Tang, Bin Wang, Zhiping Zhao, Xiaoming Xie, Weidong Wei

**Affiliations:** 1 Department of Breast Oncology, Sun Yat-Sen University Cancer Center, State Key Laboratory of Oncology in South China, Collaborative Innovation Center for Cancer Medicine, Guangzhou, China; 2 Institute of Life Science, Chongqing Medical University, Chongqing, China; 3 Institute of Hepatopancreatobiliary Surgery, Southwest Hospital, Third Military Medical University, Chongqing, China; University of North Carolina at Chapel Hill School of Medicine, UNITED STATES

## Abstract

Previous studies have indicated that carcinoembryonic antigen (CEA) and cancer antigen 15–3 (CA15-3) levels are both independent prognostic factors in breast cancer. However, the utility of CEA and CA15-3 levels as conventional cancer biomarkers in patients with triple-negative breast cancer (TNBC) remains controversial. The current study was performed to explore the predictive value of pre-therapeutic serum CEA and CA15-3 levels, and nomograms were developed including these serum cancer biomarkers to improve the prognostic evaluation of TNBC patients. Pre-therapeutic CA15-3 and CEA concentrations were measured in 247 patients with stage I–IV TNBC. Kaplan-Meier analysis showed that TNBC patients with high levels of both CEA and CA15-3 had shorter overall survival (OS) and disease-free survival (DFS) rates than those in the low-level groups (*p*<0.05). Multivariate analysis suggested that pre-therapeutic CA15-3 and CEA levels are independent predictive elements for OS (*p* = 0.022 and *p* = 0.040, respectively) and DFS (p = 0.023 and p = 0.028, respectively). In addition, novel nomograms were established and validated to provide personal forecasts of OS and DFS for patients with TNBC. These novel nomograms may help physicians to select the optimal treatment plans to ensure the best outcomes for TNBC patients.

## Introduction

Triple-negative breast cancer (TNBC) is a hypotype of breast cancer that is immunohistochemically based on the negative expression of the hormone receptors estrogen receptor (ER) and progesterone receptor (PR) and on the negative amplification of HER2 amplification[1]. Although the incidence of TNBC only accounts for a small proportion (10–17%) of all breast cancers, most TNBC patients are diagnosed with higher lymph node metastasis and mortality risk than patients with other types of breast cancer in the first five years[2–4]. Because of the absence of the expression of HER2 or ER and PR, chemotherapy is the only treatment choice for patients with TNBC[5]. However, once resistance to chemotherapy drugs occurs, the loss of life quality and sustained upward mortality rate of malignant patients will be out of control. Therefore, it is necessary to ascertain safe and practical evaluation indicators to assist both short-term and long-term treatment decisions of TNBC patients to improve survival rates. Recently, numerous studies have reported the opposite effects of some elevated blood biochemical values[6–9] and the predictive significance of pre-operative levels of carcinoembryonic antigen (CEA) and cancer antigen 15–3 (CA15-3)[10–13] in different tumor populations.

In particular, the predictive effect of pre-operative CEA and CA15-3 levels in breast cancer has gained increasing attention. Pre-operative CEA and CA15-3 levels may offer valuable information for the prognosis of breast cancer[14–16]. However, the predictive significance of these levels in breast cancer remains ambiguous due to the limitation of the number of cases[13,16,17]. Recently, nomograms have been shown to provide more precise individualized disease-related risk estimations compared to the traditional TNM staging systems[18,19]. Nomograms provide a visual representation of the regression equation and could help physicians to better utilize sophisticated statistical results. However, there is a lack of related literature providing accurate predictive nomograms of CEA and CA15-3, which are common clinical hematology indexes. Therefore, the objective and significance of this study were to inquire into the prognostic roles of pre-therapeutic CEA and CA15-3 levels by building a nomogram for resected TNBC based on known traditional clinicopathological prognostic factors.

## Materials and Methods

### Patients and methods

Clinical analysis was performed for 247 female patients, and all of them were definitively diagnosed with triple-negative breast cancer and were treated with modified radical mastectomy at the Sun Yat-sen University Cancer Center (SYSUCC) in Guangzhou, China, between January 2004 and December 2009. The ethics boards of Sun Yat-sen University Cancer Center granted ethical approval (NO.YB2016-002-03), and all patients provided written information consent. The inclusion criteria were as follows: clear pathological reports of TNBC, with no prior pre-operative anti-cancer treatments before the collection of autologous whole blood and serum tumor marker data. The exclusion criteria were as follows: (1) patients with coexisting cancers; (2) initial records of blood biochemical tests after treatment; (3) active infectious or other autoimmune disorders; (4) people without follow up; and (5) the lack of other necessary information.

### Clinical data collection

The medical records were evaluated by electronic chart review, and each patient’s medical history, age, BMI, menopausal status, and main pathological information (such as tumor size, lymph node status, hormonal status, HER2 status, histological grade, and laboratory data) were obtained. The clinical typing and staging of the malignant tumor were identified by the TNM staging system according to the AJCC (American Joint Committee on Cancer Classification, 7th edition, http://www.cancerstaging.org). Triple-negative breast cancer, just as its name implies, was confirmed by ER-, PR-, HER2- status. The absence of hormone receptor expression was stipulated based on the positive staining for ER and/or PR in less than 10% of cancerous cell nuclei, and the state of HER2 was defined according to the ASCO guidelines. Two hundred thirty patients (93.1%) underwent adjuvant chemotherapy, and 52 patients (21.1%) received adjuvant radiotherapy treatment.

### Hematological parameters

The serum tumor marker levels of CEA and CA 15–3 were obtained using an automatic electrochemistry luminescence immunoassay system (ROCHE E170; Roche, Germany). The cut-off values of CEA and CA15-3 by the X-tile program were 6.0 ng/ml and 21.8 U/ml, respectively. Additionally, the value was considered to be high or low by comparing results with the cut-off value.

### Follow-up and study endpoints

In the first 3 years, the patients were followed up by telephone every 3 months and then every 1-year until relapse or death. The day of the acquisition of definitive pathological results was defined as the initial day of follow-up, and the last follow-up date was November 27, 2015 for all of the available patients. The primary observation endpoints of the study were disease-free survival (DFS), and overall survival (OS). Disease-free survival was estimated from the date of the acquisition of definitive pathological results to the date of local recurrence or distant metastasis, death, or new neoplasms. Overall survival was estimated from the date of the acquisition of definitive pathological results to death or the date of the last follow-up.

### Statistical analysis

The optimal cut-off points for the serum cancer biomarkers of survival were determined by the minimum P value from log-rank X^2^ statistics using the X-tile 3.6.1 software (Yale University, New Haven, CT, USA)[20]. Statistical analyses were performed using SPSS 20.0 (SPSS, Chicago, IL, USA). The correlation between the patients’ characteristics and pre-therapeutic serum biomarkers was assessed by unpaired t-test or one-way analysis of variance (ANOVA), and deviations between the proportions were tested using the chi-squared test. It is essential to investigate the survival analyses and differences between the groups by the Kaplan-Meier method and log-rank test. The independent variables related to OS and DFS were confirmed using univariate and multivariate analyses. All of the statistically significant variables in univariate analyses were incorporated into multivariate analyses, and variables with a *P* >0.05 were eliminated. According to the results of the multivariable analysis, nomograms were established respectively by R 3.2.4 (http://www.r-project.org) using the *survival and rms* package[21]. The capability of the model for prognosis was judged by Harrell’s concordance index (C-index). The upper bound of the c-index is 1.0, forecasting an ideal differentiation, whereas 0.5 represents only half of the chance to correctly differentiate the outcome. Calibration curves of the nomograms for the 5-year OS and DFS were implemented by collating the prognostic survival and actual survival after error correction. *P* values less than 0.05 were considered statistically significant.

## Results

### Patient characteristics

**Fig 1:**
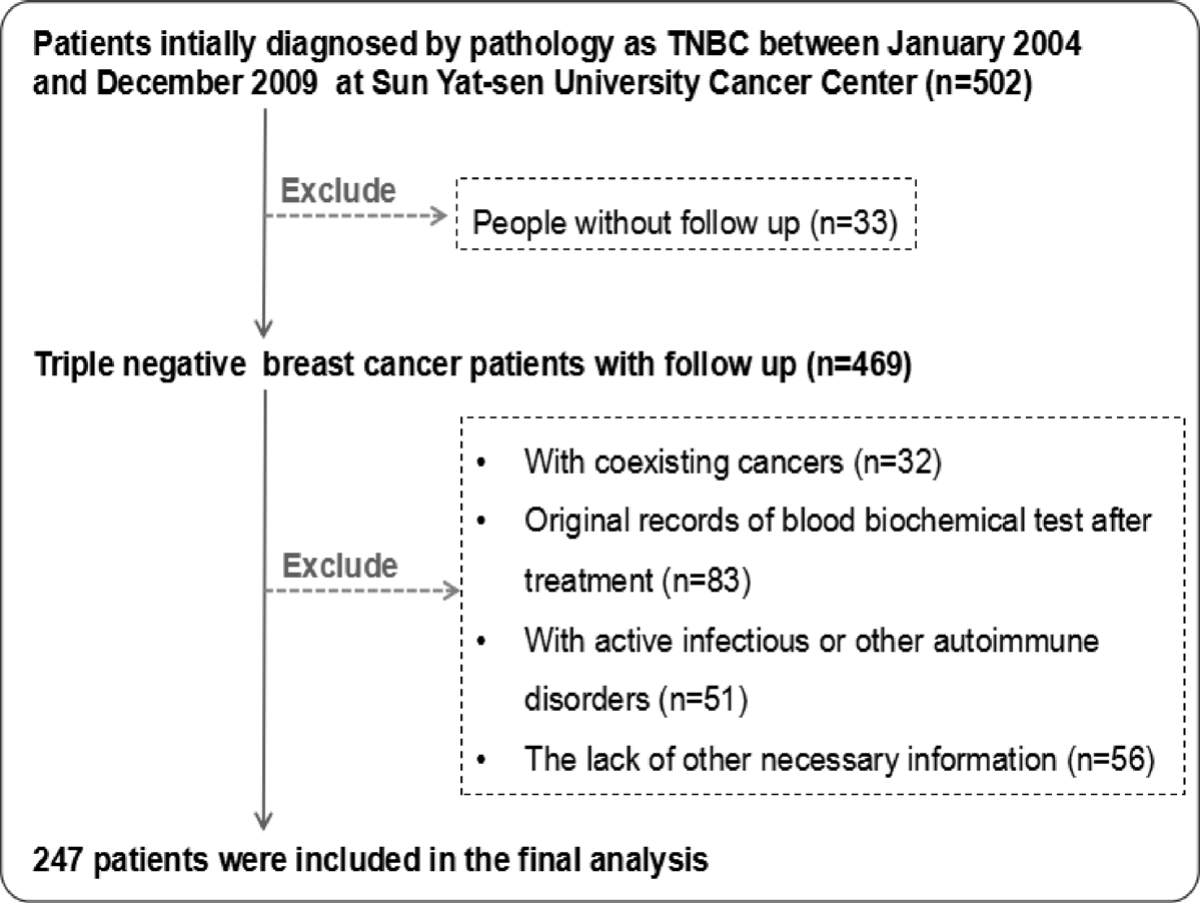
Flow chart of patient selection.

In total, 247 female patients who were pathologically confirmed as having TNBC were incorporated after qualification review from January 2004 to December 2009. The screening process is given in Fig 1. Among the 247 breast cancer patients, 108 (43.7%) developed recurrence, and 104 patients (42.1%) died during a median of 84 months (range: 2–141 months) follow-up time. The pathological classification of the 210 cases (85.0%) was invasive ductal carcinoma (S1 Table). The median age of the patients was 46.8 years (range: 22–79 years), and 163 (66.0%) patients were younger than 50 years of age. The patient characteristics and correlation between pre-therapeutic CEA and CA153 levels and clinicopathological variables of TNBC cases are displayed in Table 1. The mean value of the pre-therapeutic CEA level was 8.83±54.67 ng/ml, and it was correlated with tumor status, N status, and TNM staging (all *p* < 0.05). The mean value of the CA15-3 level was 23.89±45.40 U/ml, and it was correlated with tumor status, N status, TNM staging, and histological grade (all *p* < 0.05). In addition, patients with a high level of tumor status, TNM staging, and histological grade showed a higher CA15-3 level (*p* < 0.05). Other characteristics were not correlated with pre-therapeutic levels of the tumor markers (*p* > 0.05).

**Table 1:** Correlation between pre-therapeutic CEA and CA153 levels and clinicopathological variables of TNBC cases.

Variables	Cases (n = 247)	CEA (ng/ml)		Patients, n (%)			CA15-3(U/ml)		Patients, n (%)		
		Mean±SD	P^a^	Low CEA (< = 6.0)	High CEA (>6.0)	P^b^	Mean±SD	P^a^	Low CA15-3 (< = 21.8)	High CA15-3 (>21.8)	P^b^
**Age (years)**			0.434			0.266		0.526			0.783
< = 50	163	6.88 ±37.35		149(91.4%)	14(8.6%)		25.21±51.98		125(76.7%)	38(23.3%)	
>50	84	12.64±78.19		73(86.9%)	11(13.1%)		21.33±28.73		66(78.6%)	18(21.4%)	
**Gender**											
Female	247	8.84±54.67		222(89.9%)	25(10.1%)		23.89±45.40		191(77.3%)	56(22.7%)	
**Menopause**			0.383			0.806		0.774			0.302
no	103	12.43±74.41		92(89.3%)	11(10.7%)		24.87±57.37		83(80.6%)	20(19.4%)	
yes	104	6.27±34.30		130(90.3%)	14(9.7%)		23.19±34.60		108(75.0%)	36(25.0%)	
**BMI**			0.933			0.992		0.235			0.089
< = 25	186	9.03±59.86		167(89.8%	19(10.2%)		21.93±33.10		149(80.1%)	37(19.9%)	
>25	59	8.50±35.31		53(89.8%)	6(10.2%)		30.03±72.05		41(69.5%)	18(30.5%)	
**Tumor status**			0.082			**0.001** *		**<0.001** *			**<0.001** *
T1	69	2.08±1.06		69(100.0%)	0(0.0%)		21.09±6.43		63(91.3%)	6(8.7%)	
T2	147	11.39±67.58		128(87.1%)	19(12.9%)		23.02±32.36		111(75.5%)	36(24.5%)	
T3	15	3.17±3.10		12(80.0%)	3(20.0%)		34.27±54.13		10(66.7%)	5(33.3%)	
T4	16	19.81±64.18		13(81.2%)	3(18.8%)		73.01±131.74		7(43.8%)	9(56.2%)	
**N status**			**<0.049** *			**0.018** *		0.085			**0.002** *
0	127	2.21±2.23		121(95.3%)	6(4.7%)		17.55±28.81		108(85.0%)	19(15.0%)	
1	71	24.11±100.72		60(84.5%)	11(15.5%)		29.28±67.71		54(76.1%)	17(23.9%)	
2	39	4.26±5.38		32(82.1%)	7(17.9%)		36.34±41.93		22(56.4%)	17(43.6%)	
3	10	2.42±2.12		9(90.0%)	1(10.0%)		17.55±10.67		7(70.0%)	3(30.0%)	
**TNM Staging**			0.261			**0.010** *		**0.001** *			**<0.001** *
I	52	1.98±1.08		52(100.0%)	0(0.0%)		11.72±5.93		48(92.3%)	4(7.7%)	
II	131	11.77±71.41		116(88.5%)	15(11.5%)		21.14±33.33		104(79.4%)	27(20.6%)	
III	56	8.23±34.57		47(83.9%)	9(16.1%)		37.04±74.74		36(64.3%)	20(35.7%)	
IV	8	9.65±20.58		7(87.5%)	1(12.5%)		56.01±62.05		3(37.5%)	5(63.5%)	
**Histological grade**			0.384			0.055		**0.013** *			**0.006** *
G1/G2	124	5.82±35.82		116(93.5%)	9(6.5%)		16.75±21.82		105(84.7%)	19(15.3%)	
G3	123	11.88±68.66		106(86.2%)	17(13.8%)		31.09±59.77		86(69.9%)	37(30.1%)	
**Adjuvant chemotherapy**			0.692			0.685		0.532			0.930
no	17	3.75±5.33		15(88.2%)	2(11.8%)		17.23±10.84		13(76.5%)	4(23.5%)	
yes	230	9.21±56.63		207(90.0%)	23(10.0%)		24.38±46.93		178(77.4)	52(22.6%)	
**Adjuvant radiotherapy**			0.541			0.157		0.943			0.231
no	195	9.94±61.17		178(91.3%)	17(8.7%)		23.81±48.63		154(70.0%)	41(21.0)	
yes	52	4.71±12.68		44(84.6%)	8(15.4%)		24.20±30.74		37(71.2%)	15(28.8%)	

* p < 0.05, statistically significant.

G1: well differentiated; G2: moderately differentiated; G3: poorly differentiated

T1: ≤ 2 (cm); T2: 2< but ≤5 (cm); T3: >5 (cm); T4: invasion of chest wall and skin

N0: no regional lymph node metastasis; N1: metastasis involving 1–3 lymph nodes; N2: metastasis involving 4–9 lymph nodes; N3: metastasis involving ≥10 lymph nodes

a Using t test or ANOVA, P < 0.05 was considered statistically significant.

b Using Chi-squared test, P < 0.05 was considered statistically significant.

Abbreviations: TNBC: triple-negative breast cancer, BMI: body mass index, N status: node status, TNM: a certain stage comes from the comprehensive assessment of tumor status, regional lymph node status and metastasis status, CEA: carcinoembryonic antigen, CA15-3: cancer antigen 15–3.

### Cut-off value of pre-therapeutic CEA and CA15-3 levels

**Fig 2:**
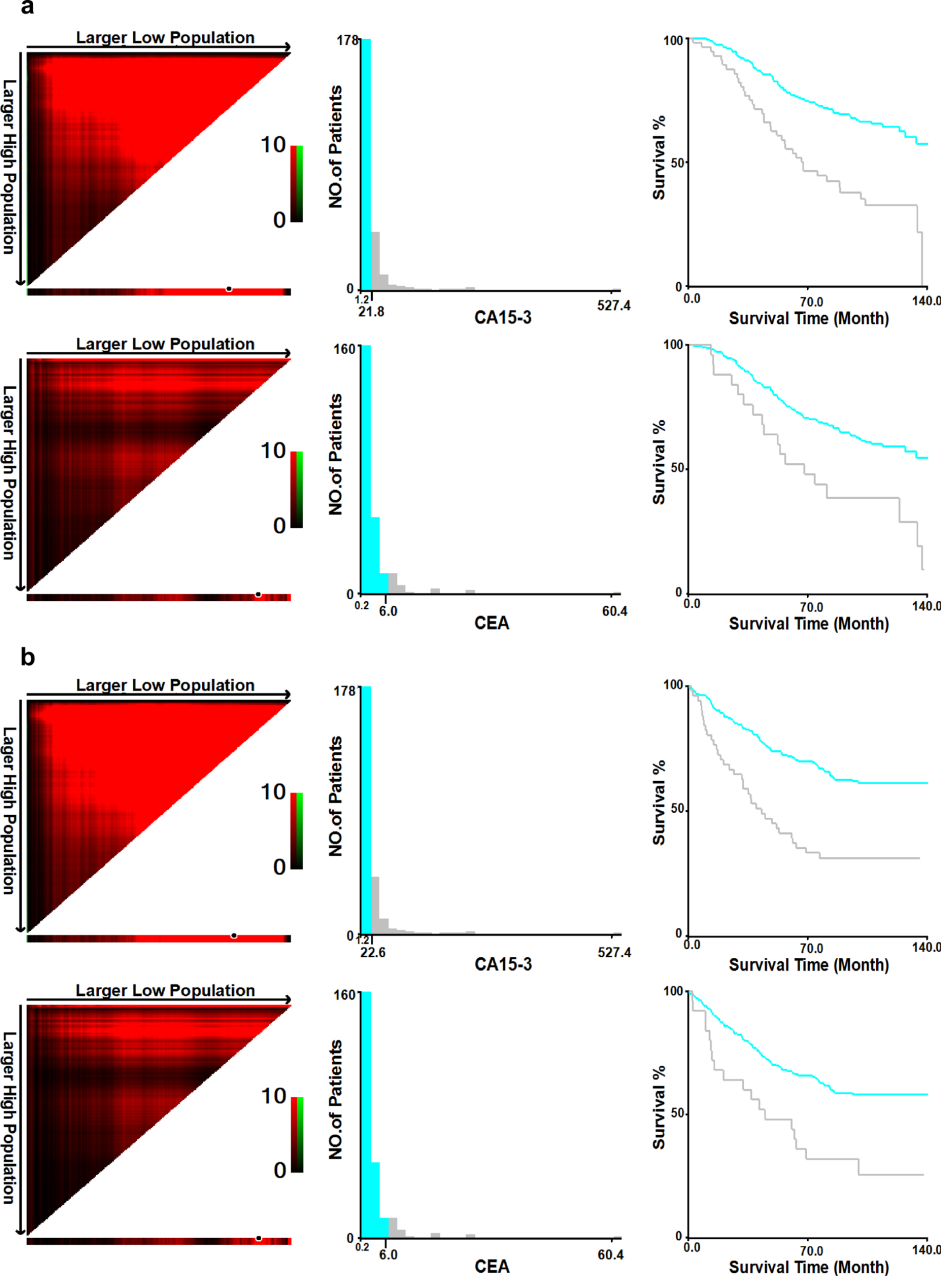
Identification of optimal cut-off values to define the CEA and CA15-3 levels as high or low.

**Fig 3:**
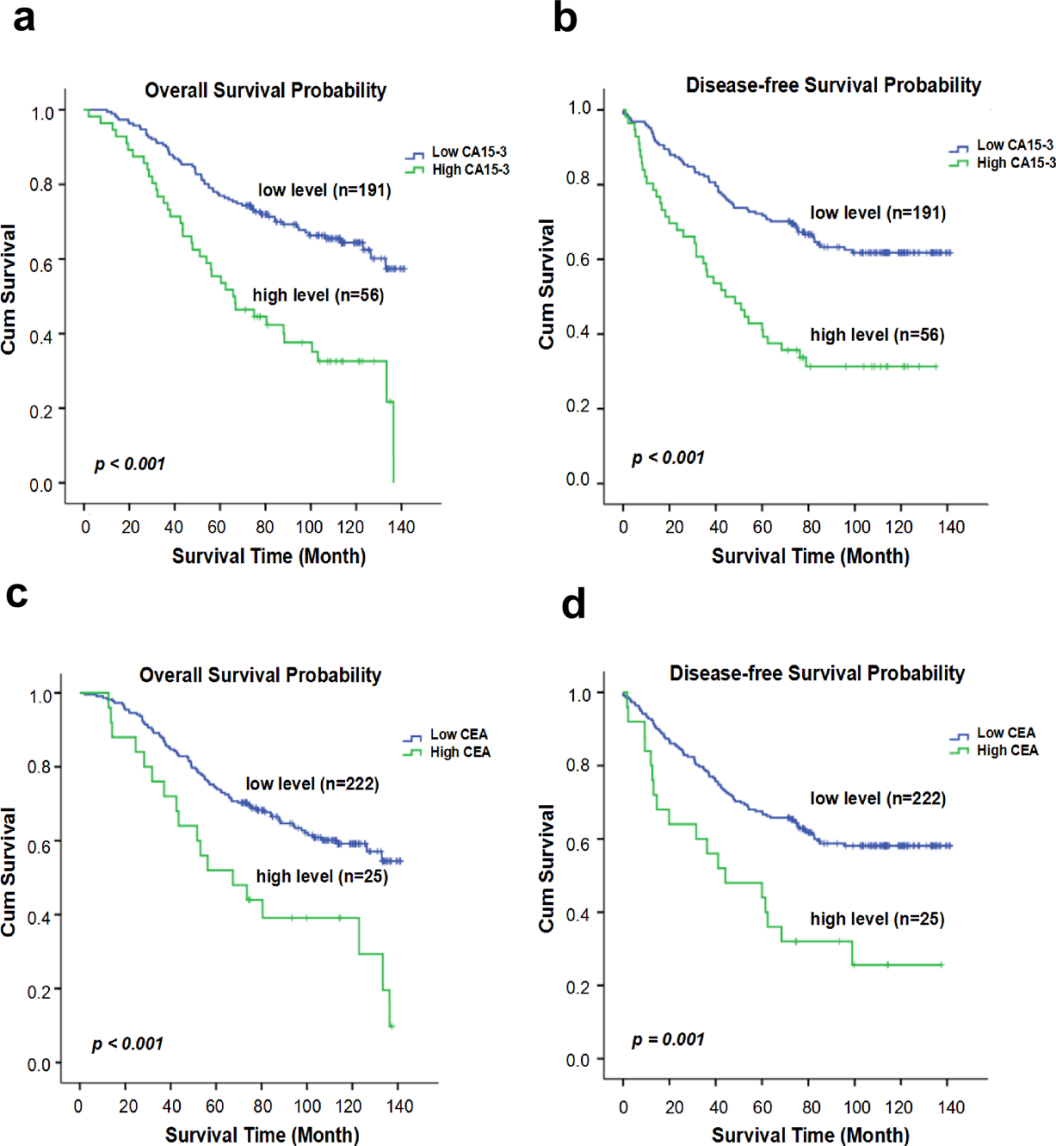
Kaplan–Meier evaluation of the probability of survival for patients with TNBC according to the serum markers in the classification. (a and b). The overall survival (OS) rate and disease-free survival (DFS) rate of the TNBC patients at different CA15-3 levels. (c and d). The overall survival (OS) rate and disease-free survival (DFS) rate of the TNBC patients at different CEA levels.

The optimal cut-off values of the pre-therapeutic CEA and CA15-3 levels for OS forecasting were identified as 6.0 ng/ml and 21.8 U/ml, respectively, using the X-tile program (Fig 2A). The χ^2^ log-rank values of CEA and CA15-3 were 10.96 (*p* = 0.024) and 23.93 (*p* < 0.001), respectively. The patients were grouped according to the cut-off values for further study (CEA ≤ 6.0 ng/ml and > 6.0 ng/ml; CA15-3 ≤ 21.8 U/ml and > 21.8 U/ml). Kaplan-Meier survival analysis revealed that CEA > 6.0 ng/ml and CA15-3 > 21.8 U/ml were remarkably associated with reduced OS and DFS (*P* < 0.001) (Fig 3). As before, the cut-off values of the CEA and CA15-3 levels for DFS were also calculated as 6.0 ng/ml and 22.6 U/ml, respectively, using the X-tile program (Fig 2B), with log-rank values of 12.54 (*p* = 0.012) and 23.75 (*p* < 0.001), respectively. The cut-off values of OS were applied to the latter analysis to maintain the consistent criterion and avoid confusion because of the minor difference between DFS and OS.

X-tile analysis was accomplished based on statistics drawn from the patient records, which were evenly divided into two groups: a training group and a validation group. The plans of the training groups are displayed in the left column figures, with graphs of the corresponding validation groups displayed in the smaller inset. The optimal separation marked by the black dot in the left column figures is exhibited as a histogram (middle column figures) and a Kaplan-Meier curve (right column figures). P values were calculated by adopting the dividing point shown in the training group and using it in the validation group as follows: (a) The optimal cut-off values for the CEA and CA15-3 levels regarding OS were 6.0 ng/ml (χ^2^ = 10.96, *p* = 0.024) and 21.8 U/ml (χ^2^ = 23.93, *p* < 0.001), respectively; (b) The optimal cut-off values for the CEA and CA15-3 levels regarding DFS were 6.0 ng/ml (χ^2^ = 12.54, *p* = 0.012) and 22.6 U/ml (χ^2^ = 23.75, *p* < 0.001), respectively.

### Survival and prognostic value of the CEA and CA15-3 levels

The 10-year OS and DFS rates were 56.0% and 54.0%, respectively, for all 247 patients, and the average survival times were 102.0 (95% CI: 96.106–108.003) months and 94.3 (95% CI: 87.448–101.173) months, respectively (S1 Fig). As the Kaplan-Meier curve implied, high CEA and CA15–3 concentrations had shorter OS and DFS rates than those in the low-level groups of TNBC patients (Fig 3 and S2 Table). For TNBC patients, the negative correlation of pre-therapeutic CEA (HR: 2.293; 95% CI: 1.377–3.816; *P* = 0.001) and CA15-3 (HR: 2.627; 95% CI: 1.759–3.923; *P* < 0.001) levels with OS were significant, along with other parameters, such as the tumor status, N status, TNM staging, and histological grade, in the univariate analysis (Table 2). Similarly, the univariate analysis showed that a worse DFS was associated with high CEA (HR: 2.412; 95% CI: 1.452–4.007; *P* = 0.001) and CA15-3 (HR: 2.586; 95% CI: 1.738–3.847; *P* < 0.001) levels along with other parameters, including tumor status, N status, TNM staging, and histological grade (Table 2). Multivariate analysis indicated that traditional clinicopathological factors (such as tumor status, N status or histological grade) were also shown to have independent prognostic value regarding OS or DFS, but the overall TNM stage lost the independent prognostic value. Moreover, CA15-3 and CEA remained as independent predictive indexes for OS and DFS (*P* < 0.05) in multivariate analysis (Table 3).

**Table 2:** Univariate COX regression analysis for Overall Survival and Disease-free Survival in patients with TNBC.

Variables	Overall Survival			Disease-free Survival		
	HR	(95% CI)	p value	HR	(95% CI)	p value
**Age (years) (**≤50 vs>50)	1.253	0.846–1.858	0.261	1.341	0.912–1.972	0.136
**Menopause** (no vs yes)	0.998	0.676–1.471	0.990	0.943	0.644–1.379	0.762
**Tumor status**						
T1	reference			reference		
T2	1.676	1.003–2.801	0.049*	1.890	1.135–3.146	0.014*
T3	2.593	1.173–5.736	0.019*	2.909	1.315–6.433	0.008*
T4	5.570	2.736–11.340	<0.001*	6.090	2.994–12.388	<0.001*
**N status**						
0	reference			reference		
1	1.691	1.043–2.744	0.033*	1.449	0.905–2.322	0.123
2	3.958	2.406–6.513	<0.001*	3.787	2.335–6.141	<0.001*
3	6.571	3.134–13.777	<0.001*	5.140	2.484–10.637	<0.001*
**TNM staging** (I-II vs III-IV)	3.260	2.197–4.837	<0.001*	3.091	2.103–4.543	<0.001*
**Histological grade** (G1/G2 vs G3)	2.150	1.438–3.214	<0.001*	1.995	1.351–2.945	0.001*
**CEA** (High/Low)	2.293	1.377–3.816	0.001*	2.412	1.452–4.007	0.001*
**CA15-3** (High/Low)	2.627	1.759–3.923	<0.001*	2.586	1.738–3.847	<0.001*

* p < 0.05, statistically significant.

T1: ≤ 2 (cm); T2: 2< but ≤5 (cm); T3: >5 (cm); T4: invasion of the chest wall and skin.

N0: no regional lymph node metastasis; N1: metastasis involving 1–3 lymph nodes; N2: metastasis involving 4–9 lymph nodes; metastasis involving N3: ≥10 lymph nodes; TNM: a certain stage comes from the comprehensive assessment of tumor status.

Abbreviations: TNBC: triple-negative breast cancer, OS: overall survival, DFS: disease-free survival, HR: hazard ratio, 95% CI: 95% confidential interval.

**Table 3:** Multivariate COX regression analysis for Overall Survival and Disease-free Survival in patients with TNBC.

Variables	Overall Survival			Disease-free Survival		
	HR	(95% CI)	p value	HR	(95% CI)	p value
**Tumor status**						
T1	reference			reference		
T2	1.165	0.673–2.014	0.585	1.406	0.821–2.409	0.215
T3	1.073	0.429–2.684	0.880	1.490	0.599–3.702	0.391
T4	2.592	0.979–6.861	0.055	4.473	1.640–12.201	0.003*
**N status**						
0	reference			reference		
1	1.259	0.743–2.133	0.392	1.072	0.643–1.785	0.790
2	2.622	0.969–7.101	0.058	3.763	1.352–10.474	0.011*
3	6.654	2.059–21.497	0.002*	7.858	2.342–26.364	0.001*
**Histological grade** (G1/G2 vs G3)	1.584	1.033–2.429	0.035*	1.498	0.994–2.256	1.498
**TNM staging** (I-II/III-IV)	0.951	0.348–2.596	0.922	0.609	0.215–1.724	0.350
**CEA** (High/Low)	1.765	1.027–3.035	0.040*	1.836	1.066–3.161	0.028*
**CA15-3** (High/Low)	1.694	1.077–2.664	0.022*	1.672	1.072–2.609	0.023*

* p < 0.05, statistically significant.

T1: ≤ 2 (cm); T2: 2< but ≤5 (cm); T3: >5 (cm); T4: invasion of the chest wall and skin.

N0: no regional lymph node metastasis; N1: metastasis involving 1–3 lymph nodes; N2: metastasis involving 4–9 lymph nodes; N3: metastasis involving ≥10 lymph nodes; TNM: a certain stage comes from the comprehensive assessment of tumor status.

Abbreviations: TNBC: triple-negative breast cancer, OS: overall survival, DFS: disease-free survival, HR: hazard ratio, 95% CI: 95% confidential interval.

### Formulation and Verification of Nomograms for Prognostic Estimation of TNBC Patients

**Fig 4:**
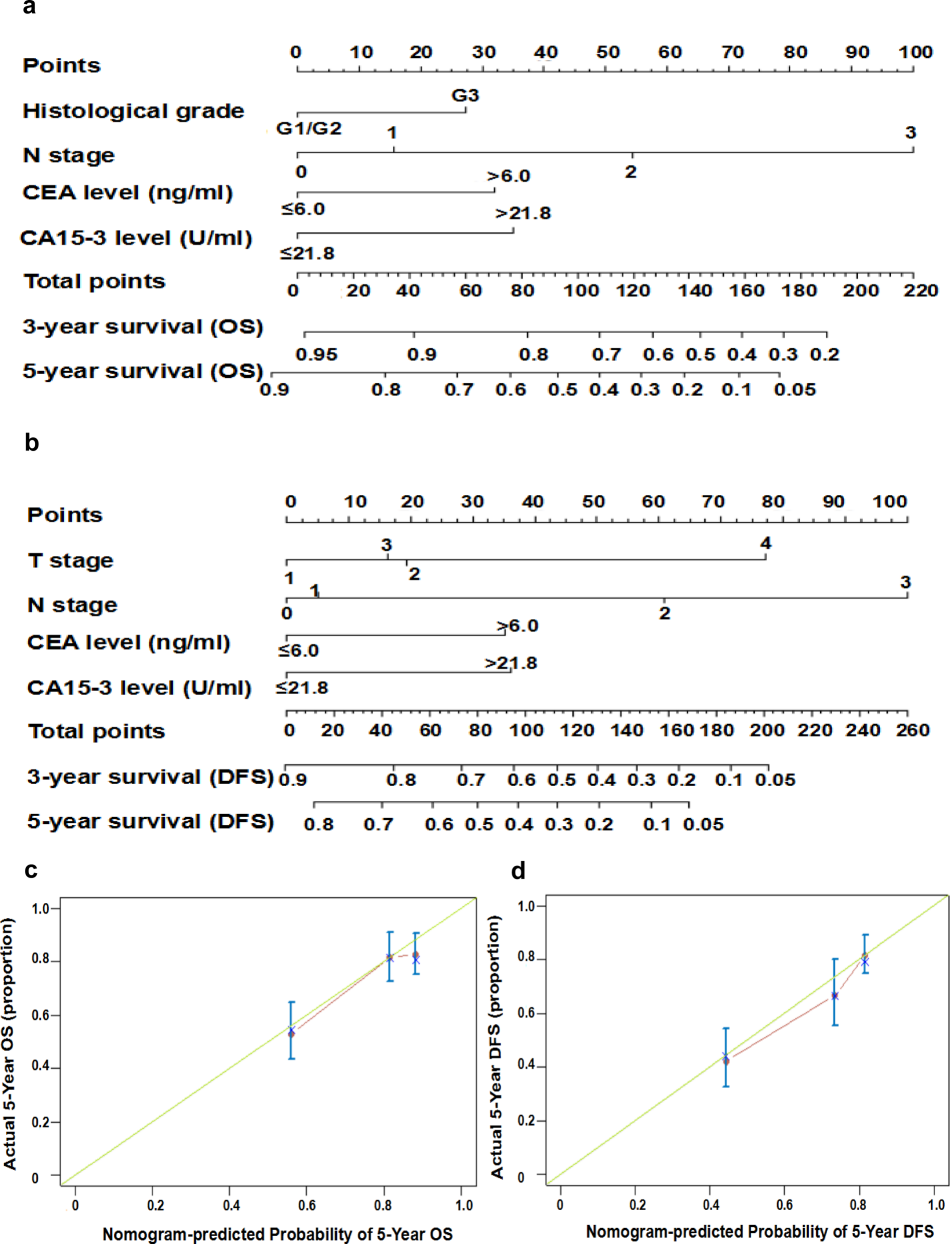
Prognostic Nomograms for patients with TNBC to predict OS and DFS. (a and b). Nomograms predict the OS and DFS of patients with TNBC via the clinicopathological characteristics and pretreatment serum cancer biomarkers. The Harrell’s c-indexes of the OS and DFS evaluation were 0.664 (95% CI: 0.613–0.714) and 0.673 (95% CI: 0.626–0.720), respectively. (c and d). Calibration graphs for the 5-year OS and DFS are shown. The x-axis represents the nomogram-forecasted chance of survival, and the y-axis represents the survival rate. The imaginary line is diagonal and shows ideal matching.

To evaluate the OS and DFS of patients with TNBC, nomograms were built based on notable independent elements for OS and DFS in the multivariate Cox regression model (Fig 4A and 4B). Nomograms can be understood by summing up each parameter score, which is shown at the top of the scale. Finding the corresponding point within the axis of total points and drawing a perpendicular line to the axis of survival probability (such as the 3-year survival or 5-year survival), the point of intersection is the patient's 3-year or 5-year odds of survival and relapse for an individual. For example, for OS, values for Histological grade of 3, N-stage 2, CEA >6 and CA15-3 >21.8 will obtain a total score of approximately 148 which means the probability for 3-year survival is 0.45 and that for 5-year survival is 0.15. The C-index values of the OS and DFS forecast were 0.664 (95% CI: 0.613–0.714) and 0.673 (95% CI: 0.626–0.720), respectively. The calibration curves for the two nomograms (Fig 4C and 4D) presented the acceptability and conformance in the original cohort between the nomogram forecast and actual observation for the 5-year OS or DFS.

### Risk-stratified groups based on the Nomogram within each TNM stage

**Fig 5:**
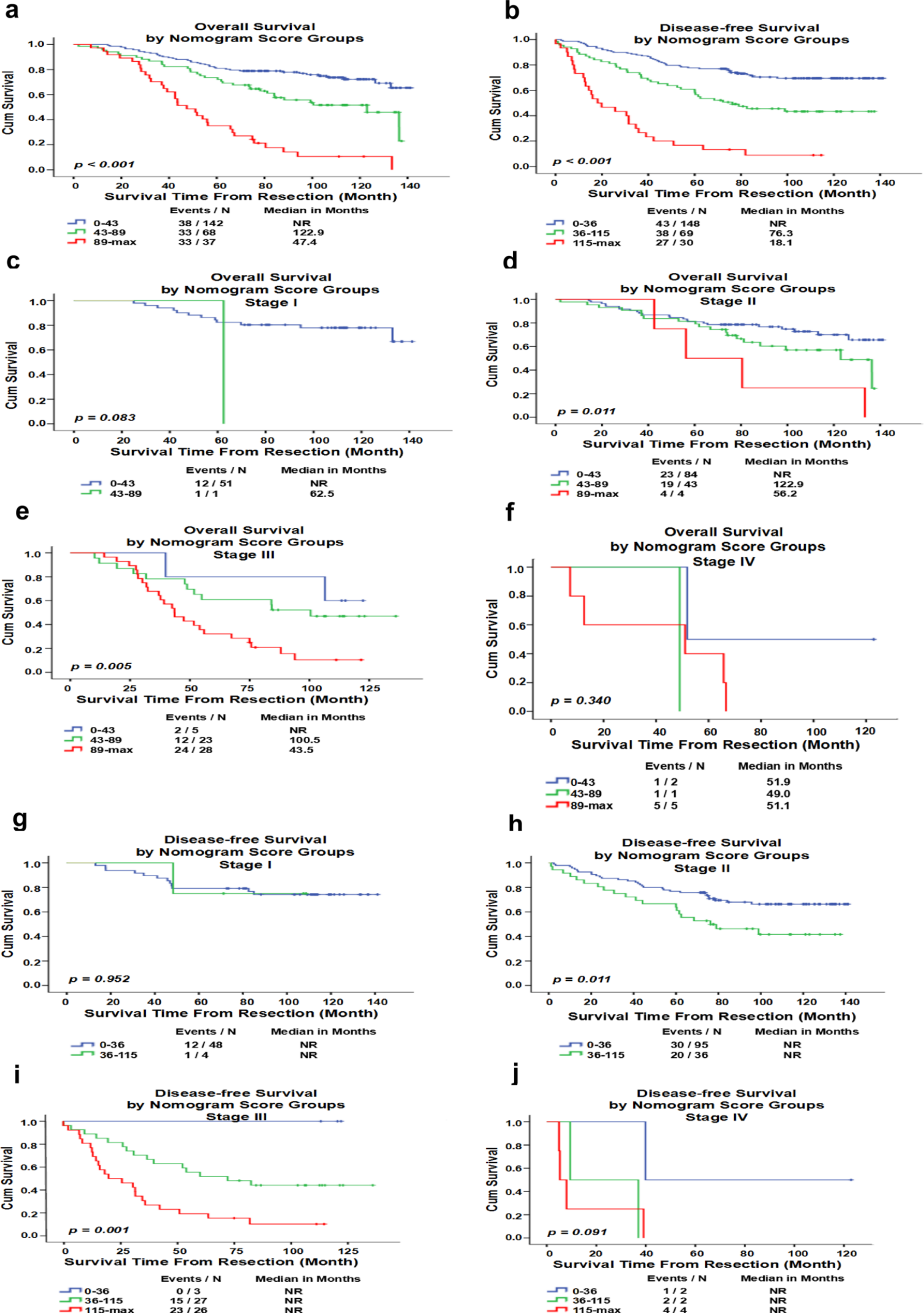
Risk group stratification Analysis At Each TNM stage. (a and b). The OS and DFS of all patients with TNBC in the different score groups. (c-j). The OS and DFS of patients with TNBC at different stages. Only subgroups with more than 10 patients are shown in the graphs. NR, not reached.

In addition to numerically contrasting the discriminating power by the C-index, the independent discriminating power of the nomogram preceding standard TNM staging was also illustrated. By dividing the TNBC patients into three distinct subgroups based on the total scores (score of OS from the nomogram: 0 to 43, 43 to 89, and ≥89; score of DFS: 0–36, 36–115, and≥115), each group corresponded to a distinct prognosis (S3 Table). After applying the cut-off values of OS or DFS to group the TNBC patients, stratification analysis of distinct subgroups showed remarkable differences between the Kaplan-Meier survival curves within each TNM stage (Fig 5).

## Discussion

The predictive values of serum cancer biomarkers have been documented for several types of cancer, including breast cancer[22,23]. Clinical stages and molecular classification are the two primary factors of current therapeutic decision-making and forecasts of the prognosis of breast cancer. Nevertheless, this classification does not satisfy the medical need for a specific subgroup. Additional biomarkers are urgently required to guide treatment and estimate the prognosis[24,25]. In current research, our multivariate analysis revealed that CEA and CA15-3 were independently negative predictive determinants for both OS and DFS in TNBC patients. However, TNM staging did not show any prognostic value for both DFS and OS, probably because the powerful prognostic ability of CEA and CA15-3 influenced the predictive function of TNM staging in addition to the tumor status, N status, and histological grade in the multivariate analysis (Table 3). Furthermore, the nomograms, which incorporate proper risk factors according to multivariate analysis, enables the doctor to obtain a better personalized estimation of the prognosis in routine clinical practice. Thus, nomograms may be better than the traditional TNM staging systems in several cancers[26]. Validation of the nomogram is indispensable to avoid over-fitting of the matrix and identify its universality[27]. In the present study, the calibration map displayed first-rank consistency between the forecast and actual observation, supporting the reproducibility and dependability of the created nomogram. Subsequently, we adopted 3 cut-off points for the OS and DFS, which showed more prognostic accuracy than classic TNM staging (Fig 5).

TNBC is an invasive phenotype that carries a worse prognosis than other luminal tumors[28,29] and represents a subclass of breast cancers with diverse clinical characterization and outcome, significant hazards, molecular characteristics and response to therapy[30]. However, serum markers may offer useful information about the phenotype of breast cancer at the early stage, when the acquisition of tissue specimens is not available in some cases[31]. Despite these supposed advantages, the associations between pre-therapeutic marker levels and prognosis in TNBC have not been elaborately investigated yet. Shao et al.[15] and Park et al.[13] showed a negative prognostic role of the CEA and CA15-3 levels in 432 and 740 breast cancer patients, respectively, with few data analyses of TNBC. However, increasingly more serum biomarkers were indicated as prognostic factors, and few nomograms were published to improve the management of patients with TNBC. In clinical research, a remarkable correlation was found between symptomatic metastasis and high CEA and CA15-3 concentrations in breast cancer patients[32]. Furthermore, our study observed that patients with high-level markers showed worse outcomes than those with low levels (S2 Table). Because serum markers are relatively convenient and inexpensive to obtain, routine inspection of their levels could offer some valuable feedback for the accurate forecast of outcomes.

As shown in several studies[15,33], higher levels of preoperative serum tumor markers represent tumor burden, which is linked to the tumor size and lymph node metastasis and predicts poorer survival in breast cancer. As expected, distinctly higher tumor biomarker levels were noted in the tumor status and TNM staging, suggesting a relationship between high levels of tumor biomarkers and tumor load (Table 1). Because the level of CA15-3 was closely correlated with the metastasis of the venous and lymphatic vasculature[34], a high CA 15–3 concentration at the beginning could predict a worse breast cancer outcome[35]. In particular, the prognostic implication of TNM staging was completely ruled out in the multivariate analysis with the incorporation of other classical predictive elements such as tumor status and nodal status (Table 3).

On account of lacking organ and tumor specificity and low sensitivity, the use of tumor markers for therapeutic decisions was generally invalidated[36]. Nevertheless, other studies have suggested that the pre-operative level of tumor markers could be helpful in association with other indicators to judge whether additional treatment should be executed[31,37], and our nomogram scoring system could help physicians to handle such issues. Furthermore, higher levels may imply a greater possibility of recurrence, and a study assessing preemptive therapy upon incremental tumor marker concentrations displayed better outcomes than the controls[38]. In addition, it has been suggested that biochemical assessment may lead to saving almost 50% of expenses compared with detection by clinical diagnostic standards, which usually require costly medical imaging techniques, such as magnetic resonance imaging[39,40]. To our knowledge, this study is the first to combine nomograms with frequently used tumor markers for the prognostic assessment of TNBC patients. Both clinicians and patients could obtain a personalized prognosis prediction after surgery via this convenient assessment method. Moreover, high-level pre-therapeutic serum tumor markers may be advantageous in estimating high-risk groups and in guiding subsequent therapy, for which the aforementioned speculation may be changed.

We must acknowledge the limitations in our retrospective study. On the one hand, our study relied exclusively on a single-institutional database, although eligibility criteria were formulated to minimize the selective bias. On the other hand, the accuracy of our nomograms should be assessed by external validation, which would help evaluate whether our nomograms may be appropriate for a new population and then generalized to other populations.

In general, we built novel nomograms to forecast the OS and DFS of patients with TNBC. With the help of this model, physicians may consider the proper utilization of the pre-therapeutic serum levels of CEA and CA15-3 to more effectively predict the survival rate of patients and discern subgroups of patients who should undergo a specific treatment strategy if necessary. In addition, if the pre-therapeutic serum CEA and CA15-3 levels could be combined with other efficient molecular factors (e.g., BRCA2 mutation) to provide further prognostic information, it may be beneficial in treatment implementation. In the meantime, further clinical trials, including a perspective cohort study, are required to illustrate and improve the validity of this model in the therapeutic decision-making field for breast cancer.

## Supporting Information

S1 Fig Kaplan–Meier estimates of the probability of survival for patients with triple-negative breast cancer. (a). The overall survival (OS) rate of 247 TNBC patients. (b). The disease-free survival (DFS) rate of 247 TNBC patients.
(TIF)

S1 Table Pathological Classification. (DOC)

S2 Table CEA and CA153 levels in TNBC patients by Kaplan-Meier survival analysis (log-rank test). (DOC)

S3 Table Point Assignment and Prognostic Score. (DOC)

## References

[1] Dai X, Li T, Bai Z, Yang Y, Liu X, Zhan J, et al Breast cancer intrinsic subtype classification, clinical use and future trends. American journal of cancer research. 2015;5(10):2929–43. . 26693050 PMC4656721

[2] Haffty BG, Yang Q, Reiss M, Kearney T, Higgins SA, Weidhaas J, et al Locoregional relapse and distant metastasis in conservatively managed triple negative early-stage breast cancer. Journal of clinical oncology: official journal of the American Society of Clinical Oncology. 2006;24(36):5652–7. 10.1200/JCO.2006.06.5664 . 17116942

[3] Dent R, Trudeau M, Pritchard KI, Hanna WM, Kahn HK, Sawka CA, et al Triple-negative breast cancer: clinical features and patterns of recurrence. Clinical cancer research: an official journal of the American Association for Cancer Research. 2007;13(15 Pt 1):4429–34. 10.1158/1078-0432.CCR-06-3045 . 17671126

[4] Reis-Filho JS, Tutt AN. Triple negative tumours: a critical review. Histopathology. 2008;52(1):108–18. 10.1111/j.1365-2559.2007.02889.x . 18171422

[5] Minami CA, Chung DU, Chang HR. Management options in triple-negative breast cancer. Breast cancer: basic and clinical research. 2011;5:175–99. 10.4137/BCBCR.S6562 . 21863131 PMC3153117

[6] Bozkurt O, Karaca H, Berk V, Inanc M, Ocak Duran A, Ozaslan E, et al Predicting the role of the pretreatment neutrophil to lymphocyte ratio in the survival of early triple-negative breast cancer patients. Journal of BUON: official journal of the Balkan Union of Oncology. 2015;20(6):1432–9. . 26854438

[7] Basu S, Harris H, Larsson A, Vasson MP, Wolk A. Is There Any Role for Serum Cathepsin S and CRP Levels on Prognostic Information in Breast Cancer? The Swedish Mammography Cohort. Antioxidants & redox signaling. 2015;23(16):1298–302. 10.1089/ars.2015.6404 . 26079659

[8] Wen J, Yang Y, Ye F, Huang X, Li S, Wang Q, et al The preoperative plasma fibrinogen level is an independent prognostic factor for overall survival of breast cancer patients who underwent surgical treatment. Breast. 2015;24(6):745–50. 10.1016/j.breast.2015.09.007 . 26482138

[9] Krenn-Pilko S, Langsenlehner U, Stojakovic T, Pichler M, Gerger A, Kapp KS, et al An elevated preoperative plasma fibrinogen level is associated with poor disease-specific and overall survival in breast cancer patients. Breast. 2015;24(5):667–72. 10.1016/j.breast.2015.08.003 . 26346586

[10] Molina R, Barak V, van Dalen A, Duffy MJ, Einarsson R, Gion M, et al Tumor markers in breast cancer- European Group on Tumor Markers recommendations. Tumour biology: the journal of the International Society for Oncodevelopmental Biology and Medicine. 2005;26(6):281–93. 10.1159/000089260 . 16254457

[11] Chen Y, Zheng YH, Lin YY, Hu MH, Chen YS. [Clinical and prognostic significance of preoperative serum CA153, CEA and TPS levels in patients with primary breast cancer]. Zhonghua zhong liu za zhi [Chinese journal of oncology]. 2011;33(11):842–6. . 22335950

[12] Zou D, Zhang B, Ba J, Ye Q, Wu G. [Expression and significance of CEA and CA153 in pleural fluid of patients with lung cancer]. Zhongguo fei ai za zhi = Chinese journal of lung cancer. 2006;9(4):337–9. 10.3779/j.issn.1009-3419.2006.04.08 . 21176450

[13] Park BW, Oh JW, Kim JH, Park SH, Kim KS, Kim JH, et al Preoperative CA 15–3 and CEA serum levels as predictor for breast cancer outcomes. Annals of oncology: official journal of the European Society for Medical Oncology / ESMO. 2008;19(4):675–81. 10.1093/annonc/mdm538 . 18037623

[14] Tang S, Zhou F, Sun Y, Wei L, Zhu S, Yang R, et al CEA in breast ductal secretions as a promising biomarker for the diagnosis of breast cancer: a systematic review and meta-analysis. Breast cancer. 2016 10.1007/s12282-016-0680-9 . 26898373

[15] Shao Y, Sun X, He Y, Liu C, Liu H. Elevated Levels of Serum Tumor Markers CEA and CA15-3 Are Prognostic Parameters for Different Molecular Subtypes of Breast Cancer. PloS one. 2015;10(7):e0133830 10.1371/journal.pone.0133830 . 26207909 PMC4514648

[16] Chu WG, Ryu DW. Clinical significance of serum CA15-3 as a prognostic parameter during follow-up periods in patients with breast cancer. Annals of surgical treatment and research. 2016;90(2):57–63. 10.4174/astr.2016.90.2.57 . 26878012 PMC4751146

[17] Wu SG, He ZY, Zhou J, Sun JY, Li FY, Lin Q, et al Serum levels of CEA and CA15-3 in different molecular subtypes and prognostic value in Chinese breast cancer. Breast. 2014;23(1):88–93. 10.1016/j.breast.2013.11.003 . 24291374

[18] Wang Y, Li J, Xia Y, Gong R, Wang K, Yan Z, et al Prognostic nomogram for intrahepatic cholangiocarcinoma after partial hepatectomy. Journal of clinical oncology: official journal of the American Society of Clinical Oncology. 2013;31(9):1188–95. 10.1200/JCO.2012.41.5984 . 23358969

[19] Liang W, Zhang L, Jiang G, Wang Q, Liu L, Liu D, et al Development and validation of a nomogram for predicting survival in patients with resected non-small-cell lung cancer. Journal of clinical oncology: official journal of the American Society of Clinical Oncology. 2015;33(8):861–9. 10.1200/JCO.2014.56.6661 . 25624438

[20] Camp RL, Dolled-Filhart M, Rimm DL. X-tile: a new bio-informatics tool for biomarker assessment and outcome-based cut-point optimization. Clinical cancer research: an official journal of the American Association for Cancer Research. 2004;10(21):7252–9. 10.1158/1078-0432.CCR-04-0713 . 15534099

[21] Frank E HJ, Rms: Regression Modeling Strategies. R Package version 3.4–0.

[22] Tomita M, Ayabe T, Chosa E, Nakamura K. Postoperative Serum CEA Level is a More Significant Prognostic Factor than Post/Preoperative Serum CEA Ratio in Non-small Cell Cancer Patients. Asian Pacific journal of cancer prevention: APJCP. 2015;16(17):7809–12. . 26625802 10.7314/apjcp.2015.16.17.7809

[23] Wu SG, He ZY, Ren HY, Yang LC, Sun JY, Li FY, et al Use of CEA and CA15-3 to Predict Axillary Lymph Node Metastasis in Patients with Breast Cancer. Journal of Cancer. 2016;7(1):37–41. 10.7150/jca.13090 . 26722358 PMC4679379

[24] Melichar B. Laboratory medicine and medical oncology: the tale of two Cinderellas. Clinical chemistry and laboratory medicine. 2013;51(1):99–112. 10.1515/cclm-2012-0496 . 22987835

[25] Malyguine AM, Strobl SL, Shurin MR. Immunological monitoring of the tumor immunoenvironment for clinical trials. Cancer immunology, immunotherapy: CII. 2012;61(2):239–47. 10.1007/s00262-011-1148-6 . 22080408 PMC11028845

[26] Sternberg CN. Are nomograms better than currently available stage groupings for bladder cancer? Journal of clinical oncology: official journal of the American Society of Clinical Oncology. 2006;24(24):3819–20. 10.1200/JCO.2006.07.1290 . 16864852

[27] Iasonos A, Schrag D, Raj GV, Panageas KS. How to build and interpret a nomogram for cancer prognosis. Journal of clinical oncology: official journal of the American Society of Clinical Oncology. 2008;26(8):1364–70. 10.1200/JCO.2007.12.9791 . 18323559

[28] O'Brien KM, Cole SR, Tse CK, Perou CM, Carey LA, Foulkes WD, et al Intrinsic breast tumor subtypes, race, and long-term survival in the Carolina Breast Cancer Study. Clinical cancer research: an official journal of the American Association for Cancer Research. 2010;16(24):6100–10. 10.1158/1078-0432.CCR-10-1533 . 21169259 PMC3029098

[29] Rakha EA, El-Rehim DA, Paish C, Green AR, Lee AH, Robertson JF, et al Basal phenotype identifies a poor prognostic subgroup of breast cancer of clinical importance. European journal of cancer. 2006;42(18):3149–56. 10.1016/j.ejca.2006.08.015 . 17055256

[30] Brouckaert O, Wildiers H, Floris G, Neven P. Update on triple-negative breast cancer: prognosis and management strategies. International journal of women's health. 2012;4:511–20. 10.2147/IJWH.S18541 . 23071421 PMC3469230

[31] Di Gioia D, Dresse M, Mayr D, Nagel D, Heinemann V, Stieber P. Serum HER2 in combination with CA 15–3 as a parameter for prognosis in patients with early breast cancer. Clinica chimica acta; international journal of clinical chemistry. 2015;440:16–22. 10.1016/j.cca.2014.11.001 . 25444743

[32] Bahrami A, Mortazavizadeh MR, Yazdi MF, Chamani M. Serial tumour markers serum carcinoembryonic antigen and cancer antigen 15–3 assays in detecting symptomatic metastasis in breast cancer patients. Eastern Mediterranean health journal = La revue de sante de la Mediterranee orientale = al-Majallah al-sihhiyah li-sharq al-mutawassit. 2012;18(10):1055–9. . 23301361 10.26719/2012.18.10.1055

[33] Molina R, Auge JM, Escudero JM, Filella X, Zanon G, Pahisa J, et al Evaluation of tumor markers (HER2/neu oncoprotein, CEA, and CA 15.3) in patients with locoregional breast cancer: prognostic value. Tumour biology: the journal of the International Society for Oncodevelopmental Biology and Medicine. 2010;31(3):171–80. 10.1007/s13277-010-0025-9 . 20361287

[34] Canizares F, Sola J, Perez M, Tovar I, De Las Heras M, Salinas J, et al Preoperative values of CA 15–3 and CEA as prognostic factors in breast cancer: a multivariate analysis. Tumour biology: the journal of the International Society for Oncodevelopmental Biology and Medicine. 2001;22(5):273–81. . 11553856 10.1159/000050627

[35] Shering SG, Sherry F, McDermott EW, O'Higgins NJ, Duffy MJ. Preoperative CA 15–3 concentrations predict outcome of patients with breast carcinoma. Cancer. 1998;83(12):2521–7. . 9874458

[36] Bast RC Jr, Ravdin P, Hayes DF, Bates S, Fritsche H Jr, Jessup JM, et al 2000 update of recommendations for the use of tumor markers in breast and colorectal cancer: clinical practice guidelines of the American Society of Clinical Oncology. Journal of clinical oncology: official journal of the American Society of Clinical Oncology. 2001;19(6):1865–78. . 11251019 10.1200/JCO.2001.19.6.1865

[37] Duffy MJ, Duggan C, Keane R, Hill AD, McDermott E, Crown J, et al High preoperative CA 15–3 concentrations predict adverse outcome in node-negative and node-positive breast cancer: study of 600 patients with histologically confirmed breast cancer. Clinical chemistry. 2004;50(3):559–63. 10.1373/clinchem.2003.025288 . 14726467

[38] Nicolini A, Carpi A, Michelassi C, Spinelli C, Conte M, Miccoli P, et al "Tumour marker guided" salvage treatment prolongs survival of breast cancer patients: final report of a 7-year study. Biomedicine & pharmacotherapy = Biomedecine & pharmacotherapie. 2003;57(10):452–9. . 14637388 10.1016/j.biopha.2003.09.004

[39] Robertson JF, Whynes DK, Dixon A, Blamey RW. Potential for cost economies in guiding therapy in patients with metastatic breast cancer. British journal of cancer. 1995;72(1):174–7. . 7599049 10.1038/bjc.1995.297 PMC2034118

[40] Delea T, McKiernan J, Brandman J, Edelsberg J, Sung J, Raut M, et al Retrospective study of the effect of skeletal complications on total medical care costs in patients with bone metastases of breast cancer seen in typical clinical practice. The journal of supportive oncology. 2006;4(7):341–7. . 16892696

