# Supplementary figures and images for: Nomograms for Predicting the Prognostic Value of Pre-Therapeutic CA15-3 and CEA Serum Levels in TNBC Patients
Source: PLoS One. 2016 Aug 25;11(8):e0161902. doi: 10.1371/journal.pone.0161902 (PMC4999206; doi:10.1371/journal.pone.0161902)

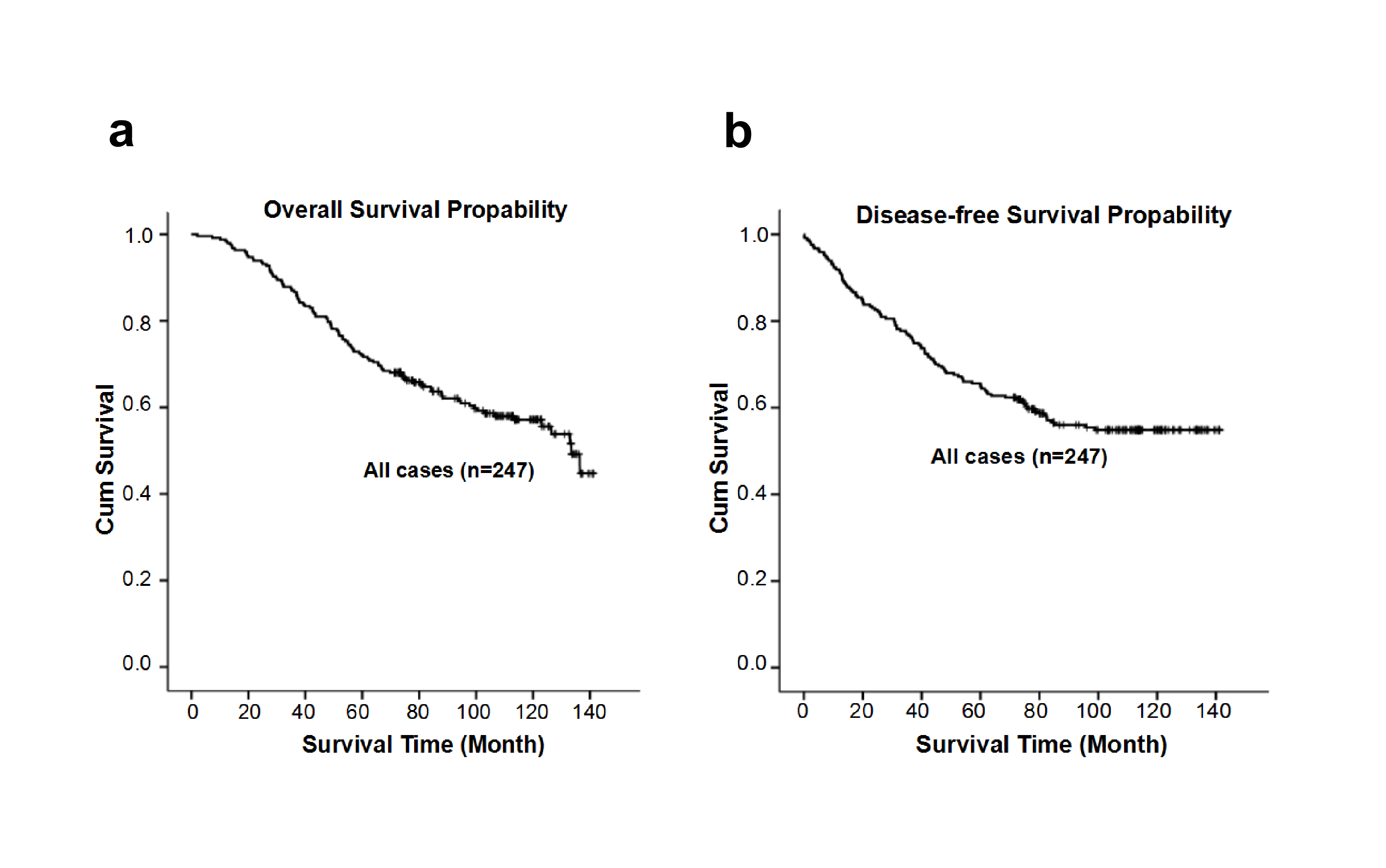

Supplement: S1 Fig — (a). The overall survival (OS) rate of 247 TNBC patients. (b). The disease-free survival (DFS) rate of 247 TNBC patients. (TIF) [file pone.0161902.s001.tif]
