# Supplementary material for: Nomograms for Predicting the Prognostic Value of Pre-Therapeutic CA15-3 and CEA Serum Levels in TNBC Patients
Source: PLoS One. 2016 Aug 25;11(8):e0161902. doi: 10.1371/journal.pone.0161902 (PMC4999206; doi:10.1371/journal.pone.0161902)
Supplement: S1 Table — (DOC) [file pone.0161902.s002.doc]

| **Table S1. Pathological Classification** | | |
| --- | --- | --- |
|  | Number | Percent(%) |
| **Invasive ductal carcinoma** | 210 | 85.0 |
| **Invasive lobular carcinoma** | 2 | 0.8 |
| **DCIS** | 2 | 0.8 |
| **LCIC** | 19 | 7.7 |
| **Microinvasive carcinoma** | 4 | 1.6 |
| **Medullary carcinoma** | 7 | 2.8 |
| **Mucinous carcinoma** | 3 | 1.2 |
| Abbreviation:DCIS:ductal carcinoma in situ; LCIC: lobular carcinoma in situ | | |
|
