# Supplementary material for: Nomograms for Predicting the Prognostic Value of Pre-Therapeutic CA15-3 and CEA Serum Levels in TNBC Patients
Source: PLoS One. 2016 Aug 25;11(8):e0161902. doi: 10.1371/journal.pone.0161902 (PMC4999206; doi:10.1371/journal.pone.0161902)
Supplement: S2 Table — (DOC) [file pone.0161902.s003.doc]

| **Table S2. CEA, CA153 level in TNBC patients by Kaplan-Meier survival analysis (log-rank test)** | | | | | | | |
| --- | --- | --- | --- | --- | --- | --- | --- |
| **Variable** | **Case** | **OS (months)** | | | **DFS (months)** | | |
|  |  | **Mean** | **Median** | **P-value** | **Mean** | **Median** | **P-value** |
| Total | 247 |  |  |  |  |  |  |
| **CEA** |  |  |  | **0.001*** |  |  | **<0.001*** |
| Low expression | 222 | 105.15 | NR |  | 97.87 | NR |  |
| High expression | 25 | 77.79 | 67.4 |  | 61.53 | 44.23 |  |
| **CA15-3** |  |  |  | **<0.001*** |  |  | **<0.001*** |
| Low expression | 191 | 109.4 | NR |  | 102.76 | NR |  |
| High expression | 56 | 77.11 | 65.73 |  | 63.78 | 44.23 |  |
| * p < 0.05, statistically significant. Abbreviation: TNBC: triple negative breast carcinoma; DFS: disease free survival; OS: overall survival; NR: not reached; CEA:carcinoembryonic antigen; CA15-3:cancer antigen 15-3 ; | | | | | | | |
|  |  |  |  |  |  |  |  |
|  |  |  |  |  |  |  |  |
