# Supplementary material for: Nomograms for Predicting the Prognostic Value of Pre-Therapeutic CA15-3 and CEA Serum Levels in TNBC Patients
Source: PLoS One. 2016 Aug 25;11(8):e0161902. doi: 10.1371/journal.pone.0161902 (PMC4999206; doi:10.1371/journal.pone.0161902)
Supplement: S3 Table — (DOC) [file pone.0161902.s004.doc]

| **Table S3. Point Assignment and Prognostic Score** | | | | | | |
| --- | --- | --- | --- | --- | --- | --- |
| Overall Survival | | |  | Disease-free Survival | | |
| Variable and Prognostic Score | Score | Estimated 5-Year Overall Survival (%) |  | Variable and Prognostic Score | Score | Estimated 5-Year Disease-free Survival (%) |
| **Histology grade** |  |  |  | **Tumor grade** |  |  |
| G1/G2 | 0 |  |  | 1 | 0 |  |
| G3 | 27 |  |  | 2 | 19 |  |
| **Nodal grade** |  |  |  | 3 | 16 |  |
| 0 | 0 |  |  | 4 | 77 |  |
| 1 | 16 |  |  | **Nodal grade** |  |  |
| 2 | 54 |  |  | 0 | 0 |  |
| 3 | 100 |  |  | 1 | 5 |  |
| **CEA level** |  |  |  | 2 | 61 |  |
| ≤6.0ng/ml | 0 |  |  | 3 | 100 |  |
| >6.0ng/ml | 32 |  |  | **CEA level** |  |  |
| **CA15-3 level** |  |  |  | ≤6.0ng/ml | 0 |  |
| ≤21.8U/ml | 0 |  |  | >6.0ng/ml | 35 |  |
| >21.8U/ml | 35 |  |  | **CA15-3 level** |  |  |
|  |  |  |  | ≤21.8U/ml | 0 |  |
|  |  |  |  | >21.8U/ml | 36 |  |
| **Total prognostic score*** |  |  |  | **Total prognostic score*** |  |  |
| 0-43 |  | 75 |  | 0-36 |  | 72 |
| 43-89 |  | 55 |  | 36-115 |  | 29 |
| ≥89 |  | 50 |  | ≥115 |  | 20 |
| Abbreviation: CEA:carcinoembryonic antigen, CA15-3:cancer antigen 15-3 | | | | | | |
